# Socioeconomic and sociodemographic factors related to allergic diseases in Korean adolescents based on the Seventh Korea Youth Risk Behavior Web-based Survey: a cross-sectional study

**DOI:** 10.1186/s12887-016-0549-2

**Published:** 2016-01-27

**Authors:** Kyung-Suk Lee, Yeong-Ho Rha, In-Hwan Oh, Yong-Sung Choi, Sun-Hee Choi

**Affiliations:** Department of Pediatrics, CHA Bundang Medical Center, CHA University School of Medicine, 59 Yatap-ro, Bundang-gu, Seongnam-si, Gyeonggi-do 13496 South Korea; Department of Pediatrics, Kyung Hee University School of Medicine, 23, Kyungheedae-ro, Dongdaemun-gu, Seoul 02447 South Korea; Department of Preventive Medicine, School of Medicine, Kyung Hee University, 26, Kyungheedae-ro, Dongdaemun-gu, Seoul 02447 South Korea; Department of Pediatrics, Kyung Hee University Hospital at Gangdong, 892, Dongnam-ro, Gandong-gu, Seoul 05278 South Korea

**Keywords:** Asthma, Allergic rhinitis, Atopic dermatitis, Socioeconomic factor, Adolescent, Smoking, Drinking

## Abstract

**Background:**

Various international reports have shown that socioeconomic and sociodemographic variables are correlated with allergic diseases; however, little is known about how these variables affect Korean adolescents. This study was conducted to identify socioeconomic and sociodemographic risk factors for allergic diseases in Korean adolescents to provide information for preventing and managing such conditions.

**Methods:**

Data from the 2011 Korea Youth Risk Behavior Web-based Survey (KYRBWS-VII) of 75,643 adolescents were used. An anonymously administered online survey was conducted to collect dependent variable information on perceived sexes, residence, family affluence (Family Affluence Scale; FAS), parental education levels, subjective academic achievement, obesity, drinking and smoking. The independent variables were asthma, allergic rhinitis and atopic dermatitis. Multivariate logistic regression was used to analyze the correlations between the dependent and independent variables.

**Results:**

Low subjective academic achievement, obesity, drinking and smoking were risk factors for asthma. High FAS, parental bachelor’s degree and high subjective academic achievement were risk factors for allergic rhinitis. Finally, high FAS, maternal bachelor’s degree and high subjective academic achievement were risk factors for atopic dermatitis.

**Conclusion:**

We found that high socioeconomic status (SES) was a risk factor for allergic diseases in Korean adolescents. We propose that the greater access to medical services and immunization (e.g., hygiene hypothesis) afforded by high SES influenced the prevalence of allergic diseases. Thus, as the Korean economy develops further, the prevalence of allergic diseases is likely to increase. Controlling harmful behavioral risk factors, such as drinking and smoking, may help to prevent adolescent allergic diseases.

## Background

Allergic diseases such as asthma, allergic rhinitis and atopic dermatitis are common chronic diseases in adolescents worldwide. A series of studies by the International Study of Asthma and Allergies in Childhood (ISAAC) found that the prevalence rates of these diseases were 0.8–32.6 % for asthma, 1–45 % for allergic rhinitis, and 0.2–24.6 % for atopic dermatitis [1–3]. A study conducted by ISAAC in 2010 showed a prevalence rate of 8.3 % for asthma, 29.9 % for allergic rhinitis, and 24.0 % for atopic dermatitis in 12- to 13-year-old Korean adolescents [4]. Additionally, results of the Korea National Health and Nutrition Examination Survey (KNHANES) administered in 2011 showed that the prevalence rate for asthma was 3.3 %, that for allergic rhinitis was 20.9 %, and that for atopic dermatitis was 9.8 % in 12- to 18-year- olds [5].

Allergic diseases not only deteriorate quality of life [6] but also cause a high economic burden; and Koreans spent approximately 252 billion won on treatments for asthma, 332 billion won for allergic rhinitis, and 57 billion won for atopic dermatitis in 2012 [7]. One strategy to prevent and manage allergic diseases is to identify the risk factors to which they are related. Many studies have shown the association between allergic disease prevalence rates, socioeconomic factors and genetic risk factors. Discrepancies in the prevalence rates of allergic diseases are associated with different levels of socioeconomic development [8–11]. Adolescents tend to perceive their socioeconomic status (SES) as equivalent to that of their parents; thus, bias is a problem when measuring SES accurately in surveys [12]. The family affluence scale (FAS) is a four-question questionnaire that has been implemented to measure adolescents’ SES more objectively [12].

A number of studies have shown that factors such as SES, obesity, smoking and drinking are associated with the high prevalence rates of allergic diseases [5, 7, 9, 12, 13]. Based on the results from the Davos “Global Allergy Forum” of 2011, a multidisciplinary approach to preventing and managing allergic diseases was emphasized [14]. The Atopy Asthma Education Information Center and the Atopy Asthma-Safe Schools program have been operating in Korea to create various approaches to address allergic diseases [15, 16].

One German study reported atopic dermatitis to be associated with high and middle SES, in contrast to asthma and allergic rhinoconjunctivitis [9]. Another study described a higher prevalence of allergic rhinitis and eczema in the high SES group than in the low SES group [11]. On the other hand, a systematic review reported that asthma was associated with lower SES, whereas the prevalence of allergies was related to higher SES [17]. However, no study has targeted adolescents or has focused on the relationships between prevalence rates of allergic diseases and socioeconomic and sociodemographic variables, although there are a number of studies on prevalence rates of allergic diseases in large Korean population samples [4, 18]. As studies rarely control for SES when investigating allergic diseases, little is known about risk factors for allergic diseases independent of SES in Korean adolescents.

This study was conducted to identify the relationships between allergic diseases and socioeconomic and sociodemographic variables using data from the Korea Youth Risk Behavior Web-based Survey (KYRBWS-VII), a representative measure of Korean adolescents’ health status, in order to provide information that may help modify risk factors as part of the effort to reduce allergic disease prevalence.

## Methods

### Survey methods and participants

Data for this study were drawn from the Korea Center for Disease Control and Prevention 2011 KYRBWS-VII results [13]. The KYRBWS-VII was conducted from September to October 2011 using an anonymously self-answered online survey. The KYRBWS-VII provides a student sample that is representative of the entire Korean middle school and high school student population. All middle school and high school students were defined the entire population of the KYRBWS-VII. The sample selection was carried out in three stages [13].

#### First stage: stratification

The study population was stratified by geographic region (considering size of city, number of students and number of residents) and school type (middle school, general high school or specialized high school) to minimize standard error.

#### Second stage: sample allocation

The sample was derived from 400 middle schools and 400 high schools and selected by proportional sampling to match the study population.

#### Third stage: stratified cluster sampling

The first sampling unit was schools, selected by systematic sampling and the second sampling unit was classes, sampled by randomized selection from selected schools. All students in selected classes participated in the survey except when circumstances such as absence did not permit inclusion.

Through this process, 75,643 participants (aged 13–18 years; grades 7–12) were sampled in this survey. All participants were assigned a unique identification number and answered an online questionnaire.

The KYRBWS-VII data are freely available in de-identified form through the website of KYRBWS [19].

This study was granted exemption from institutional review board (IRB) review by the Institutional Review Boards of Kyung Hee University Hospital at Gangdong (KHNMC 2014-10-003).

### Questionnaire and definition of variables

#### Definition of allergic diseases

We adopted the definitions of allergic diseases used in the Korea Center For Disease Control and Prevention’s Korea Health Statistics, Korea National Health and Nutrition Examination Survey (KNHANES V-1) [20].

To assess the prevalence of asthma, students were asked the following question: “Have you had wheezing or whistling in the chest within the last 12 months?” [1, 13, 20]. If the student answered “yes,” we defined this as asthma. To assess the prevalence of allergic rhinitis, students were asked the following question: “Have you ever been diagnosed with allergic rhinitis by a doctor?” If the student answered “yes,” we classified this as allergic rhinitis [13, 20, 21]. To assess the prevalence of atopic dermatitis, students were asked the following question: “Have you ever been diagnosed with atopic dermatitis (or eczema) by a doctor?” If the student answered “yes,” we identified this as atopic dermatitis [13, 20, 21].

#### Socioeconomic and sociodemographic status information

Residential areas were classified as follows: large cities (metropolitan area, megalopolis and some cities of Gyeonggi-do), small and medium-sized cities (all other cities excluding large cities), and districts.

Four questions, each containing scaled answers, were prepared to obtain information regarding the FAS:

Q1: Does your family own a vehicle? A1: No (0), One (1), Two or more (2); Q2: Do you have your own bedroom? A2: No (0), Yes (1); Q3: How many family trips (includes staying more than one night) have you taken in the last 12 months? A3: None (0), One (1), Two or more (2); Q4: How many computers (including laptops) does your family own? A4: None (0), One (1), Two or more (2). Students were classified into a low (0–3), middle (4–5) or high (6–7) FAS group according to the sum of the scores they checked [12]. The FAS measure was validated in the Health Behavior in School-Aged Children Survey [22] and shows strong consistency in the associations between FAS and health [23].

Parental education level was evaluated by the following question, Q: What is your parent’s education level (for each parent separately)? A: Bachelor’s degree or higher, high school graduate, middle school graduate or less.

Subjective academic achievement was determined by the following question, Q: How would you rate your academic achievement? A: High, high-middle, middle, middle-low, low.

#### Obesity

Obesity was determined by calculated body mass index (BMI; kg/m^2^), which was based on a self-administered survey. Obesity was defined if BMI was ≥ 25, or exceeded the 95th percentile of sex-specific and age-specific BMI in the 2007 Korea National Growth Chart [12, 13, 24, 25].

#### Drinking and smoking

Drinking and smoking were considered to have occurred if a student drank or smoked more than once within 1 month [13].

### Statistical analysis

Pearson’s *chi*-square test was used for the cross-tabulation analysis of asthma, allergic rhinitis and atopic dermatitis with respect to the socioeconomic and sociodemographic variables. Risk factors for asthma, allergic rhinitis and atopic dermatitis were investigated with multiple logistic regression methods. The analysis was adjusted by socioeconomic and sociodemographic variables (sex, residence, FAS, parental education level, smoking, drinking and obesity). SPSS version 21.0 software (IBM Co., Armonk, NY, USA) was used for all analyses to evaluate the stratified cluster sampling design survey. A *p-*value <0.05 was considered significant.

## Results

### Prevalence of allergic diseases (Table 1)

**Table 1:** Prevalence of allergic diseases in Korean adolescents (*n* = 75,643)

Variable	Asthma		Allergic rhinitis		Atopic dermatitis	
	Prevalence, % (S.E.)	*p-*value	Prevalence, % (S.E.)	*p* value	Prevalence, % (S.E.)	*p-*value
Total population	11.1 (0.1)		33.9 (0.3)		23.1 (0.2)	
Sex		0.02*		0.10		0.00*
Boy	10.8 (0.2)		34.2 (0.3)		20.3 (0.2)	
Girl	11.4 (0.2)		33.5 (0.3)		26.2 (0.2)	
Residence		0.71		0.00*		0.00*
District	11.9 (0.4)		26.3 (0.8)		20.8 (0.6)	
Small and Medium city	11.1 (0.2)		35.2 (0.4)		23.2 (0.3)	
Large city	11.0 (0.2)		33.4 (0.3)		23.3 (0.3)	
FAS^a^		0.09		0.00*		0.00*
Low	10.8 (0.2)		29.5 (0.4)		21.7 (0.3)	
Middle	11.0 (0.2)		33.9 (0.3)		23.1 (0.3)	
High	11.5 (0.2)		38.0 (0.4)		24.4 (0.3)	
Paternal education		0.00*		0.00*		0.00*
Middle or less	12.5 (0.5)		28.9 (0.8)		20.4 (0.7)	
High school	11.1 (0.2)		32.1 (0.4)		22.8 (0.3)	
College or higher	11.2 (0.2)		38.3 (0.4)		24.6 (0.3)	
Unknown	9.2 (0.3)		25.8 (0.5)		19.9 (0.4)	
Maternal education		0.00*		0.00*		0.00*
Middle or less	11.5 (0.5)		29.9 (0.9)		20.8 (0.7)	
High school	11.2 (0.2)		33.4 (0.3)		23.3 (0.2)	
Bachelor or higher	11.2 (0.2)		38.8 (0.4)		24.9 (0.3)	
Unknown	9.3 (0.3)		25.6 (0.5)		19.7 (0.4)	
Subjective academic achievement		0.00*		0.00*		0.00*
Low	14.4 (0.4)		28.9 (0.5)		20.6 (0.5)	
Middle-low	11.3 (0.2)		31.5 (0.4)		22.9 (0.3)	
Middle	9.9 (0.2)		33.4 (0.4)		22.5 (0.3)	
High-middle	10.8 (0.3)		37.3 (0.4)		24.5 (0.3)	
High	10.5 (0.4)		38.5 (0.6)		24.5 (0.5)	
Obesity		0.02*		0.51		0.22
(−)	10.9 (0.1)		34.0 (0.3)		23.1 (0.2)	
(+)	11.8 (0.4)		34.5 (0.7)		23.8 (0.5)	
Drinking		0.00*		0.53		0.22
(−)	9.8 (0.1)		33.9 (0.3)		23.2 (0.2)	
(+)	16.0 (0.3)		33.6 (0.5)		22.7 (0.4)	
Smoking		0.00*		0.01*		0.00*
(−)	9.9 (0.1)		34.1 (0.3)		23.4 (0.2)	
(+)	19.7 (0.5)		32.4 (0.6)		20.7 (0.4)	

^a^Family Affluence Scale

*p < 0.05

The overall prevalence rates of asthma, allergic rhinitis and atopic dermatitis were 11.1 %, 33.9 %, and 23.1 %, respectively.

The asthma prevalence rate was significantly higher in female than in male participants. This result varied depending on the parents’ education level and subjective academic achievement. The following characteristics were associated with significantly higher asthma prevalence rates: obesity (11.8 %), drinking (16.0 %), and smoking (19.7 %).

The allergic rhinitis prevalence rate did not differ between the sexes, but the prevalence rate was significantly lower in districts than in other locations. High FAS and high parental educational level were associated with significantly higher prevalence compared with the respective reference groups. The group with high subjective academic achievement had the highest prevalence rate (38.5 %) for allergic rhinitis, this rate being significantly different from that of the other groups. The prevalence rate in the non-smoking group (34.1 %) was significantly higher than that in the smoking group (32.4 %).

The prevalence rate of atopic dermatitis was significantly higher for girls than for boys (26.2 % vs. 20.3 %, respectively). Living in large, small or medium-sized cities resulted in higher prevalence rates than living in a district. The high FAS group showed the highest prevalence rate (24.4 %), which was significantly different from the other FAS groups. The highest prevalence rate was seen when paternal education level was a bachelor’s degree (24.6 %) and maternal education level was a bachelor’s degree (24.9 %). The lowest prevalence rate was seen when paternal education level was less than middle school (20.4 %).

The group with high subjective academic achievement showed the highest prevalence rate (24.5 %), and the group with low subjective academic achievement showed the lowest rate (20.6 %). The non-smoking group (23.7 %) showed a higher prevalence rate than did the smoking group (20.7 %).

### Risk factors for allergic diseases (Table 2)

**Table 2:** Adjusted logistic regression analysis of allergic diseases among Korean adolescents (*n* = 75,643)

Variable	Asthma		Allergic rhinitis		Atopic dermatitis	
	Adjusted OR	95 % CI	Adjusted OR	95 % CI	Adjusted OR	95 % CI
Sex (Boy)						
Girl	1.18*	1.13, 1.25	0.95*	0.91, 0.99	1.39*	1.34, 1.45
Residence (Districts)						
Small and Medium cities	0.94	0.85, 1.03	1.43*	1.32, 1.56	1.10*	1.02, 1.19
Large cities	0.94	0.86, 1.03	1.30*	1.20, 1.41	1.10*	1.02, 1.19
Family affluence scale (Low)						
Middle	1.05	0.99, 1.12	1.11*	1. 06, 1.16	1.04	0.99, 1.09
High	1.08	0.99, 1.16	1.25*	1.19, 1.31	1.07*	1.01, 1.12
Paternal education (Middle or less)						
High school	0.93	0.83, 1.04	1.06	0.97, 1.15	1.05	0.95, 1.16
Bachelor or higher	1.01	0.89, 1.14	1.22*	1.11, 1.34	1.09	0.99, 1.21
unknown	0.85	0.73, 0.99	0.96	0.86, 1.07	0.97	0.85, 1.10
Maternal education (Middle or less)						
High school	1.01	0.89, 1.16	1.06	0.97, 1.17	1.10	0.99, 1.22
Bachelor or higher	1.04	0.91, 1.19	1.18*	1.07, 1.30	1.16*	1.04, 1.29
unknown	0.90	0.76, 1.07	0.84*	0.75, 0.95	1.01	0.89, 1.14
Subjective academic achievement (Low)						
Middle-low	0.83*	0.77, 0.90	1.05	0.98, 1.11	1.09*	1.01, 1.17
Middle	0.74*	0.68, 0.81	1.10*	1.03, 1.17	1.04	0.97, 1.11
High-middle	0.84*	0.77, 0.92	1.26*	1.19, 1.34	1.15*	1.07, 1.24
High	0.81*	0.73, 0.89	1.29*	1.20, 1.39	1.18*	1.09, 1.29
Obesity (−)						
(+)	1.13*	1.04, 1.22	1.04	0.98, 1.11	1.13*	1.06, 1.20
Drinking (−)						
(+)	1.38*	1.30, 1.47	1.03	0.98, 1.07	1.05	1.00, 1.11
Smoking (−)						
(+)	1.89*	1.76, 2.04	1.01	0.95, 1.07	0.96	0.90, 1.02

Adjusted by sex, residence, paternal and maternal education levels, Family Affluence Scale, subjective academic achievement, obesity, drinking and smoking

*p < 0.05

An adjusted logistic regression method was used to identify risk factors for asthma, allergic rhinitis and atopic dermatitis using the variables sex, residential area, FAS, parental educational level, subjective academic achievement, obesity, drinking and smoking.

Female sex (odds ratio [OR], 1.18; 95 % confidence interval [CI], 1.13–1.25), obesity (OR, 1.13; 95 % CI, 1.04–1.22), drinking (OR, 1.38; 95 % CI, 1.30–1.47) and smoking (OR, 1.89; 95 % CI, 1.76–2.04) were significant risk factors for asthma, and high subjective academic achievement was negatively correlated with asthma.

Living in a large, small or medium-sized city, high parental education level (father OR, 1.22; 95 % CI, 1.11–1.34; mother OR, 1.18; 95 % CI, 1.07–1.30), high FAS (middle OR, 1.11; 95 % CI, 1.06–1.16; high OR, 1.25; 95 % CI, 1.19–1.31) and high subjective academic achievement (middle OR, 1.10; 95 % CI, 1.03–1.17; high-middle OR, 1.26; 95 % CI, 1.19–1.34; high OR, 1.29; 95 % CI, 1.20–1.39) were significant risk factors for allergic rhinitis.

Female sex, living in a large, small or medium-sized city, high FAS (OR, 1.07; 95 % CI, 1.01–1.12), maternal bachelor’s degree (OR, 1.16; 95 % CI, 1.04–1.29), high subjective academic achievement (middle-low OR, 1.09; 95 % CI, 1.01–1.17; high-middle OR, 1.15; 95 % CI, 1.07–1.24; high OR, 1.18; 95 % CI, 1.09–1.29) and obesity were significant risk factors for atopic dermatitis.

## Discussion

In this study, we have demonstrated that high SES is a risk factor for allergic diseases in Korean adolescents. To our knowledge, no other study has used a self-administered online survey such as the KYRBWS to investigate the relationships between socioeconomic factors and prevalence rates of allergic diseases in Korean adolescents.

Many studies have investigated the relationships between household SES and allergic diseases. The FAS is a commonly used indicator to measure SES and parental education level [12]. The relationships we found between FAS and allergic diseases were similar to those of other, similar studies [10, 26]. The middle- and high-FAS groups displayed higher ORs for allergic rhinitis than did the low-FAS group. The high-FAS group also had a significantly higher OR for atopic dermatitis than did the low-FAS group. In other words, as SES became higher on the basis of the FAS, the prevalence rate of allergic diseases increased. Parental education level was adopted as another variable to measure SES, because high SES groups tend to have higher education levels [12]. Asthma was not related to parental education level, but having parents with a bachelor’s degree was a risk factor for allergic rhinitis, and having a mother with a bachelor’s degree was a risk factor for atopic dermatitis. These results are compatible with the so-called “hygiene hypothesis,” which links the sanitary environment of Westernized areas with a higher prevalence rate of allergic diseases during the early phase of development. Children raised in a high-SES family have better access to advanced medical treatment, vaccination and adequate nutrition; thus, they are less likely to be exposed to infection [10]. Under these circumstances, the Th2 cell pathway becomes stronger than the Th1 cell pathway, which increases allergic disease prevalence rates [10]. However, children with high SES benefit from access to medical services through improved disease detection, which raises the disease diagnosis rate. Socioeconomic conditions may result in an uneven distribution of medical personnel and institutions depending on the location. Although a low SES group uses more medical services, a high SES group is in a better position to use medical services when both groups’ desire to use medical services is adjusted for [27]. Therefore, adolescents from high-SES families may have better access to medical services and may therefore present with higher prevalence rates of allergic diseases. Subjective academic achievement is a self-evaluation that represents academic achievement independent of the actual grade and may be an appropriate indicator of adolescents’ perception of their SES [12]. Therefore, the high OR for allergic rhinitis and atopic dermatitis with high parental education level, high FAS and high subjective academic achievement seems reasonable. However, no such relationships were found for asthma in this study.

High SES has been linked to older parental age and high breastfeeding rate, whereas lower SES has been associated with factors such as obesity, tobacco smoking and indoor and outdoor pollution [17, 28]. Unfortunately, there is no obvious biological mechanism to explain these associations, but a birth cohort study in England recently revealed that eliminating exposure to tobacco smoke during the maternal pregnant period can prevent the high rates of asthma in children of low SES.[17, 28] Although it is not possible to reduce all risk factors related to SES, it may be possible to reduce the prevalence of allergic diseases through further interventional studies and policies, such as anti-smoking campaigns, aimed to control such factors.

Here, sex-specific analysis showed that the asthma prevalence rate was significantly higher for girls than for boys. The 2011 KNHANES results showed that the asthma prevalence rate for boys (3.9 %) was higher than that for girls (2.7 %) in the same age group [16]. In contrast, an online survey conducted in the US in 2011 showed that the current asthma prevalence rate was higher for girls than that for boys [29]. No significant sex difference in the prevalence rate for allergic rhinitis was observed in the present study, but the OR for girls was 0.95, which was slightly lower than that for boys. Girls had a higher OR (1.38) for atopic dermatitis than did boys, consistent with the results of other domestic and international studies [3, 4, 26]. Potential reasons for the discrepancies between girls and boys are the hormone environment and the survey administrating method. A smaller discrepancy between the sexes has been a trend in more recent studies [4, 30].

No significant difference in the prevalence rate of asthma was observed in terms of residential area; however, the prevalence rates for allergic rhinitis and atopic dermatitis were significantly higher in large cities, making this a risk factor. Additionally, the ISAAC results in 1995 and 2000 dealing with children (6–12 years old) and adolescents (12–15 years old) support the conclusion that urban areas are associated with higher asthma prevalence rates than are rural areas [4]. Interestingly, the ISAAC results in 2010 with first-grade students showed that the asthma prevalence rate was higher in rural than in urban areas [4]. A recent study revealed that the asthma prevalence rate in semi-rural areas such as Andong was higher than that in Seoul, and if potentially hazardous industrial facilities were located near a residential area, the OR for asthma could increase [31]. The prevalence rates for allergic diseases are related to the level of exposure to toxic airborne chemicals such as sulfur dioxide, carbon monoxide or nitrogen dioxide [32].

The prevalence rates of allergic diseases were significantly higher in our study than in the 2011 KNHANES but were quite similar to those from the 2010 ISAAC [4]. The discrepancies in the prevalence rates are assumed to be due to the different methods used in each survey. Investigators and participants conducted personal interviews in KNHANES, whereas face-to-face conversation was not employed for the KYRBWS and ISAAC.

In this study, obesity was a risk factor for asthma and atopic dermatitis, but did not significantly increase the odds of allergic rhinitis. Obesity is a well-known risk factor for asthma [33–35]. Several studies have shown that inflammatory cells accumulate in fatty tissues, secrete cytokines and cause inflammatory effects, which result in decreased adiponectin levels and are associated with increased prevalence of both eczema and symptoms of atopic dermatitis [36, 37]. Some studies have shown a positive correlation between obesity and atopic dermatitis, consistent with our findings [33, 36]. This result suggests that appropriate obesity management may be helpful for preventing and controlling allergic diseases.

Drinking was associated with a 1.38-fold increased risk for asthma (*p* < 0.05), although it was unrelated to allergic rhinitis or atopic dermatitis. Gonzalez-Quintela et al. reported that ethanol consumption of more than 140 g/week was associated with increased prevalence of sensitization to pollen [38]. Alcohol not only damages Th1 lymphocytes but also increases Th2 lymphocytes regulating the cell-mediated immune response [39]. Alcohol consumption increases the odds of clinical manifestations of asthma and allergic rhinitis [40]; thus, it is necessary to seek ways to reduce alcohol consumption in adolescents.

Smoking was not correlated with either allergic rhinitis or atopic dermatitis, but was associated with a significantly increased odds of asthma (OR 1.88), the highest OR among all the variables. Smoking was more strongly linked to asthma in girls than in boys [41]. Adolescent smoking is correlated with asthma outbreaks and is therefore a risk factor for asthma [34]. Smoking has been demonstrated to exacerbate acute-phase Th2 cell-driven airway inflammation and to delay tolerance for inhaled allergens [42]. Therefore, deterring adolescents from smoking could potentially be an effective measure for reducing asthma outbreaks among adolescents.

We showed that obesity, alcohol consumption and smoking during adolescence can affect the frequency of allergic disease outbreaks. Obesity is significantly related to negative self-image, negative perceptions of friends, family and school interactions [43]. Therefore, parenting interventions targeting obesity prevention are important [44]. School-based intervention programs can help with smoking prevention and reducing alcohol use in adolescents [45, 46]. Good family relationships and opportunities for prosocial involvement are protective factors for adolescent problem behaviors [47, 48]. Therefore, national school and family-based programs are needed to control these risk factors and prevent allergic outbreaks.

Although the results of this study may be representative of national epidemiological cohorts, there were several limitations. First, the cross-sectional nature of the study means that the correlations between allergic diseases and risk factors do not imply causality. However, the associations were consistent with those of the ISAAC, suggesting that they are robust. Second, this study was based on a self-administered survey with some level of subjectivity, which may have created bias. For instance, adolescents tend to overestimate their heights and underestimate their weights, which can distort BMI [12]. However, because BMIs collected from both the KYRBWS and Children-Adolescent Standard Growth Chart of 2011 [25] were similar, the self-report bias was likely minimal. Third, the identification of asthma was not based on the “diagnosis from doctors” questionnaire, unlike the other two diseases. However, we used the definitions in the Korea Health Statistics, KNHANES V-1 to assess allergic diseases [20], which is also in accordance with the prevalence from Korean national statistics. In addition, because the prevalence of diseases in this study was dependent on medical providers’ diagnosis, and medical service usage differed according to SES, there may potentially have been a disparity in allergic disease diagnosis between students from low or high socioeconomic backgrounds. Finally, there may be a difference between the actual data and the self-reports of subjective academic achievement, drinking and smoking. However, the strength of our study is that the KYRBWS was conducted anonymously; therefore, participants likely answered sensitive questions, such as those regarding smoking or drinking alcohol, or obesity, more frankly. We recruited >70,000 students by sampling without bias, which may have enhanced the power of the study.

## Conclusions

Lifestyle choices such as smoking, drinking and obesity were more significant risk factors for asthma than was SES, whereas high SES was a risk factor for allergic rhinitis and atopic dermatitis. We expect that as the Korean economy improves, the prevalence rates of allergic diseases will increase, and harmful behavior (smoking and drinking) by adolescents should be controlled. Further research is needed to identify the existence of the disparity and inequality in health care among adolescents having different socioeconomic backgrounds and to control allergic disease factors related to socioeconomic status.

## Abbreviations

BMI: body mass index
CI: confidence interval
FAS: family affluence scale
ISAAC: the International Study of Asthma and Allergies in Childhood
KNHANES: the Korea National Health and Nutrition Examination Survey
KYRBWS: the Korea Youth Risk Behavior Web-based Survey
OR: odds ratio
SES: socioeconomic status
